# Prevalence and Predictors of Self-Reported Consistent Condom Usage among Male Clients of Female Sex Workers in Tamil Nadu, India

**DOI:** 10.1155/2014/952035

**Published:** 2014-06-01

**Authors:** Saumya Rastogi, Bimal Charles, Asirvatham Edwin Sam

**Affiliations:** AIDS Prevention and Control Project, Voluntary Health Services (VHS), Rajiv Gandhi Road, T.T.T.I. Post Adyar, Chennai, Tamil Nadu 113 600, India

## Abstract

Clients of female sex workers (FSWs) possess a high potential of transmitting HIV and other sexually transmitted infections from high risk FSWs to the general population. Promotion of safer sex practices among the clients is essential to limit the spread of HIV/AIDS epidemic. The aim of this study is to estimate the prevalence of consistent condom use (CCU) among clients of FSWs and to assess the factors associated with CCU in Tamil Nadu. 146 male respondents were recruited from the hotspots who reportedly had sex with FSWs in exchange for cash at least once in the past one month. Data were analyzed using bivariate and multivariate methods. Overall, 48.6 and 0.8 percent clients consistently used condoms in the past 12 months with FSWs and regular partners, respectively. Logistic regression showed that factors such as education, peers' use of condoms, and alcohol consumption significantly influenced clients' CCU with FSWs. Strategies for safe sex-behaviour are needed among clients of FSWs in order to limit the spread of HIV/AIDS epidemic in the general population. The role of peer-educators in experience sharing and awareness generation must also be emphasized.

## 1. Introduction

Clients of female sex workers (FSWs), also known as the “bridge population”, act as a bridge between the high risk group of FSWs and the general population [1]. It is estimated that in India, there are as many as 8.5 million male clients of FSWs between 15 and 49 years and of these men, 55 percent are married [2]. There is evidence to show that multiple partners and inconsistent condom use among the clients lead to the spread of sexually transmitted infections (STIs) among their other sexual partners such as wives and lovers [3–6]. Although the HIV prevalence in Tamil Nadu has been showing a declining trend with it currently being 0.38 percent as estimated by sentinel surveillance, the HIV prevalence among the clients of FSWs ranged from as high as 2 to 4.2 percent in Tamil Nadu [7, 8].

The behavioural surveillance survey (BSS) carried out in 2006 was performed to assess risk behaviour in specific population groups in India and to measure behavioural changes from BSS 2001 to BSS 2006. The report on female sex workers (FSWs) and their clients detailed the observations of the national BSS 2006 among the FSWs and their clients, which was conducted in all states and union territories of the country. Nearly 96 percent of clients of FSWs in Tamil Nadu were aware of the role of consistent condom use (CCU) in preventing HIV/AIDS, yet a much lower 58.2 percent of clients consistently used condoms with FSWs in the last three months and a mere 1.7 and 0.6 percent consistently used condoms with noncommercial nonregular partners and regular partners (spouse or live-in partner), respectively. Additionally, on average-in the three preceding months-men in Tamil Nadu had sex with 5 FSWs [1]. This inconsistent use of condom and high volume of FSW use clearly point at the risk of HIV and STIs among the clients and also their sexual partners.

In the past, a great degree of research has gone into examining the trends and determinants of the risk behaviour among the FSWs in India as well as worldwide, but the same has not been the case with the clients of FSWs despite high HIV prevalence among them [9–16]. A few studies which examined the risk behaviour among the clients of FSWs concluded that age, literacy, marital status, employment, age at first paid sex, number of FSW partners, visiting the same FSW, self-efficacy (self-efficacy relates to one's confidence in performing a behaviour, and an important aspect of this confidence is possessing the skills to execute the behavior), norms for condom use, knowledge about STIs, consumption of alcohol before visiting FSWs, and drugs during sex played a major role in predicting condom use by clients [8, 17–19]. In India, the volume of research work on the sexual risk behaviours of clients of FSWs is miniscule, with only a few studies focusing on it [8, 18, 20]. In light of the present observation, an empirical research to examine the risk behaviour of the clients of FSWs is urgently needed. With a clear understanding of the predictors, efforts can be channelized to direct interventions at the level of this bridge population. Thus the objectives of the present study are to investigate the extent of consistent condom use with FSWs and regular partners among the clients of FSWs and also to explore client related determinants of consistent condom usage with the FSWs.

## 2. Methods

The present paper utilizes data from the State Level Communication Campaign (SLCC) survey conducted in the year 2010 in Tamil Nadu state. AIDS prevention and control (APAC) project supported Tamil Nadu State AIDS Control Society (TANSACS) for undertaking the SLCC survey. Overall 10 districts were covered in the survey, namely, Tiruvallur, Tiruvannamalai, Villupuram, Cuddalore, Namakkal, Perambalur, Erode, Dindigul, Pudukkottai, and Sivagangai. The SLCC survey was carried out among five population groups—female sex workers (FSWs), men who have sex with men (MSM), clients of FSWs, vulnerable women and young men, and women with the aim of examining the risk perception of HIV/AIDS and STIs among most at risk population (MARP). Since the focus of the present paper was to examine the consistent condom usage and determinants among male clients of FSWs, only the clients of FSWs have been considered for analysis.

The clients of FSWs were recruited from the “*hotspots*” (areas within a site where there is significant concentration of HRGs (high risk groups) are referred to as “hotspots”. Within hotspots, HRGs may solicit, cruise, and interact with other HRG members or have sex or share injecting drugs). The list of hotspots to be covered in the survey was obtained from the supervisors of the respective districts. From this list, the hotspots to be surveyed were selected using simple random sampling. The selected hotspots were visited by the interviewers at specific times of the day as advised by the district supervisors. Visiting at specific hours such as “after dark” increased the interviewers' chances of encountering a large client population. The respondents were then selected from the hotspots using purposive sampling with the help of predefined inclusion criteria. Those respondents who fulfilled the inclusion criteria of “men between 18 and 49 years, recruited from commercial sex access points, and had sex with any type of FSWs in exchange for cash at least once in the past one month” were recruited for the survey. Given the sensitive nature of the study, the survey was conducted using self-administered questionnaires after obtaining informed consent from the respondents. To ensure privacy to the respondents, they were administered questionnaires at places suggested by them.

The sample size of the clients of FSWs to be recruited for the survey was calculated as follows. The total population size of clients of FSWs in Tamil Nadu was not known. The prevalence of consistent condom use (*P*) was 0.60 [1] and precision/margin of error (*d*) was ±10% at 95 percent confidence interval. The critical value of the confidence interval for standard normal distribution (*z*) was 1.96. The sample size thus calculated for the clients of FSWs was 93. A total of 150 clients were decided to be recruited to compensate for nonresponse and incomplete information. A final sample of 146 clients of FSWs was obtained after data collection which increased the precision level from 10 to 8 percent. The district-wise break-up of the sample was as follows: Tiruvallur-17, Tiruvannamalai-15, Villupuram-17, Cuddalore-13, Namakkal-19, Perambalur-9, Erode-15, Dindigul-14, Pudukkottai-14, and Sivagangai-13. The nonresponse rate was about 15%.

This survey collected information on the socioeconomic and demographic characteristics of the respondents. The participants were also asked about their awareness and perceptions regarding HIV/AIDS and other STIs, condom and lubricant usage, counseling and testing for HIV/AIDS and exposure to radio, television, newspapers and magazines, and so forth. The covariates which were examined for their association with CCU included sociodemographic knowledge and behavioural variables. These were age in years (15–24, 25–35, 36–49), years of education (0–5, 6–10, 11-12, >12), occupation (unemployed, unskilled labor, skilled labor, service, and driving), marital status (never married, married and living with spouse, widowed/divorced/separated), awareness of STIs (no, yes), knowledge of role of condom in preventing STIs (no, yes), use of condoms during sex by peers (no, yes), alcohol intake before visiting FSWs (very often, rarely, never), and exposure to television (no, yes). The dependent or the target variable in the present study was the frequency of condom use with the FSWs by clients of FSWs (inconsistent, consistent). CCU was defined as “always” using condoms and inconsistent condom use as “sometimes”/“rarely”/“never” using condoms. The reference period for the frequency of condom use by the clients was 12 months prior to the survey.

The analysis was performed using the statistical software SPSS-17. In addition to the descriptive statistics, Pearson chi-square test (*χ* ^2^) was employed to examine the association between each of the explanatory variables and CCU among clients of FSWs. In cases where the expected count in the cells was less than five, likelihood ratio and fisher's exact test were used in the bivariate analysis. Multivariate logistic regression was performed to calculate the net effects of the covariates on the frequency of condom use by clients of FSWs. *P* values of less than 0.05 were considered to be significant.

## 3. Results

### 3.1. Sample Characteristics

Out of the 146 clients of FSWs included in the study, the majority (64.4 percent) of clients of FSWs belonged to the age group of 25–35 years. The age range of the sample was 18–49 years. Most (72.6 percent) were married and living with spouse. The rest were either unmarried (24.7 percent) or divorced/separated/widowed (2.7 percent). Half of the clients of FSWs had 6–10 years of formal education. 18.5 percent and 15 percent of clients had 11-12 and even higher education, respectively. Most of the clients were skilled laborers (33.6 percent) or unskilled laborers (skilled laborers included cultivators, masons, and carpenters and unskilled laborers included domestic helps, casual/nonagricultural labourers) (24.7 percent). Some were employed in government or private service sector (16.4 percent) and as drivers of bus/truck/auto (13.7 percent). Some were also unemployed (11.6 percent) amongst the sample. With regard to alcohol consumption, majority (78.8 percent) had ever consumed alcohol in their lives and of these, 13 percent consumed it daily, 43.8 percent consumed it at least once a week, and 13.7 percent had it monthly (table not shown).

### 3.2. Prevalence of Consistent Condom Usage

More than half the clients (51.4 percent) did not use condoms consistently during sex with FSWs in the past 12 months. Of note, 7.5 percent (*n* = 11) used condoms with FSWs only “rarely” and one client had “never” used a condom with FSWs. Even more alarming are the consistent condom usage by the clients with their spouses/regular partners. Just 1 client (0.8 percent) “always” used condom with his regular partner in the preceding 12 months. Nearly 60 percent of the clients had “never” used a condom with their regular partner (Table 1).

### 3.3. Self-Reported Condom Use

Table 2 gives information on the proportion of clients using condoms consistently with the FSWs in the past 12 months by their selected background characteristics. The frequency of condom use with FSWs among clients did not vary significantly with their sociodemographic characteristics. However, the rate of CCU among the clients fairly increases with increasing years of education (*P* = 0.13). Clients' occupations also seemed to influence the use of condom among them. More unemployed, unskilled laborers, and skilled laborers used condoms consistently with FSWs as compared to the clients who were employed in private or government services and those who were drivers (*P* = 0.18). Only 25 percent of those clients who were divorced/widowed/separated used condoms consistently with FSWs as compared to nearly 58 percent of clients who were married (*P* = 0.28) Even though the knowledge variables did not cast significant effects on the CCU among the clients of FSWs a trend was observed where higher knowledge level regarding STIs was related to a higher consistent condom usage among the clients (*P* = 0.08). Among the behavioural variables, peers' use of condoms and alcohol consumption when visiting FSWs were significantly associated with CCU among the clients. Those clients who admitted that their peers used condoms during sex were more likely to consistently use condoms with FSWs (*P* = 0.00). Of importance, those clients who had never consumed alcohol when visiting an FSW had a remarkably higher rate (83.3 percent) of consistently using condoms during sex than those who consumed it (*P* = 0.004).

### 3.4. Logistic Regression Estimates of Consistent Condom Usage

In Table 3 the unadjusted and the adjusted odds ratios of consistent condom usage with FSWs by clients in the 12 preceding months are presented along with their *P* values. It is observed that in this analysis, unlike the bivariate chi-square analysis, years of education are a significant predictor of consistent condom usage among the clients of FSWs. Adjusted odds show a steady trend of increasing CCU among the clients as their years of education increase. Those clients who had 11-12 years of education were nearly 13 times more likely to use condoms consistently with the FSWs as compared to those who had only 0–5 years of education (AOR = 13.33; 95% CI = 2.07–85.81; *P* = 0.006). Peers' use of condom exercises a significant impact on the frequency of condom use by clients with both the unadjusted and adjusted odds ratios. Those clients, who were aware that their friends or acquaintances used condoms, were almost 13 times more inclined to consistently use condoms during sex with FSWs (AOR = 13.00; 95% CI = 4.94–34.25; *P* = 0.000). The results show that consumption of alcohol by clients when visiting FSWs is a major deterrent for using condoms with FSWs. It was found that those clients who never consumed alcohol when visiting FSWs were nearly 7.5 times more likely to consistently use condoms with them (AOR = 7.48; 95% CI = 1.34–41.81; *P* = 0.02). The multivariate analysis also reveals that the associations of the other explanatory variables are not significant enough to influence frequency of condom use among the clients.

## 4. Discussion

This study attempted to find the prevalence and correlates of CCU among male clients of FSWs in the past 12 months. Prevalence of CCU by the clients with the FSWs was found to be 48.6 percent which is considerably lower than the overall Indian estimate of 74 percent [1]. However, it is comparable to the prevalence of 55 percent CCU in Singapore [17]. On the other hand, studies in Sichuan province of China (30.8 percent) and Vietnam (7 percent) have shown that the prevalence of CCU was poorer as compared to the present results [21, 22]. In non-Asian countries such as Mexico, Peru, and Australia, the prevalence of CCU was moderate to high as compared to the prevalence in the present study. Mexico recorded a CCU prevalence of 49.7 percent, Peru 85.8 percent, and Australia 76.8 percent among the clients [19, 23, 24]. Additionally, the prevalence of CCU with the regular partners was considerably low at 0.8 percent and corresponds with the overall estimate for Tamil Nadu which is 0.6 percent [1]. Low CCU with regular partners has also been observed in other studies and the reasons for it ranged from condom use being equated with lack of trust on the spouse to dislike for condoms [25, 26]. This finding has indicated the increased risk of STIs that the general population may be exposed to.

In the multivariate analysis, it was observed that there was a positive association between education and CCU among the clients. Interestingly, awareness of HIV/STIs was not a significant predictor of CCU. This goes to show that often mere awareness of HIV and STIs does not translate into practicing safe sex behaviour by people. People with higher education are usually well-to-do and thus give more value to life and take precautions to not get into unprotected casual sex [27]. An Asian study too corroborated the present finding wherein education was a significant predictor of CCU among clients of Hijra sex workers [28].

Additionally, the multivariate analysis also showed that peers' use of condoms significantly influenced clients' CCU with FSWs. In India, since the use of sex work cannot be discussed within the families due to restrictive social norms, men discuss it amongst their peer network. Men identify their peers who use sex work as their equals and their use of condoms acts as a major driver to adopt safer sex practices. Several other studies as well have shown a similar association between clients' consistent use of condom and the use of condom by their peers [9, 22, 29].

This study also shows alcohol consumption by clients at the time of visiting FSWs as a significant correlate of CCU. Those who never consumed alcohol had a significantly higher likelihood of using condoms consistently with FSWs. It is a known fact that inebriation impairs decision making and propriety and may be a potential reason to not to practice safe sex behaviour. A couple of other studies have also shown that the clients who were intoxicated did not use condoms with sex workers on all occasions [17, 30]. In fact this holds true even for the FSWs' use of condoms; alcohol consumption by them is a significant predictor of noncondom use [31].

## 5. Conclusion

Clients of FSWs are undoubtedly a potential population for transmitting HIV and STIs from high risk FSWs to the low risk general population. High risk groups such as female sex workers, men who have sex with men, transgenders/Hijras, injecting drug users, and bridge population (truckers and migrants) receive the highest priority under the targeted interventions under National AIDS Control Programme (NACP), though clients of sex workers are considered as one of the key bridge populations [32]. It is essential that there should be specific strategies under the NACP to effectively target the clients of sex workers. Emphasis on consistent condom use and not just condom use, as the single most important tool for preventing HIV/AIDS, needs to be placed when devising HIV control strategies for clients of FSWs. Efforts must also be made to address the misconceptions associated with condom use with spouse/regular partners. Keeping in view that education determines CCU among the clients, it is also recommended that along with the HIV/AIDS awareness programs, adult education or literacy program may also be introduced among them. Additionally, the link between intoxication and HIV/AIDS must be made clear to the clients; suitable strategies to tackle the problem of alcohol consumption could be introduced to them. The effectiveness of behavioral interventions in improving clients' consistent condom use has been reported in various studies including a notable multicentric study by Avahan India which found that a significantly higher proportion of clients used condoms consistently as a result of the behavioural change communication campaign [33]. Thus behavioural change strategies such as peer education with proven success among the clients of FSWs must be employed.

## 6. Limitations

In spite of the best efforts to have a fair representation of the clients of FSWs in Tamil Nadu, it was not possible to recruit clients who were solicited by hidden sex workers operating out of homes and lodges. Secondly, we believe that a recall period for consistent condom use should have been shorter in our study to minimize recall bias. Nonetheless, the present study is a beginner in highlighting the problem of inconsistent condom use among the clients of FSWs and paves the way for further in-depth research.

## Figures and Tables

**Table 1:** Frequency of condom usage among clients of FSWs, with FSWs and with their regular partners in the past 12 months in Tamil Nadu.

Frequency of condom usage	Percentage (*n*)
With FSWs	
Always (consistent condom use)	48.6 (71)
Sometimes	45.3 (63)
Rarely	5.5 (8)
Never	0.7 (1)
Total	**100.0 (146)**
With regular partners	
Always (consistent condom use)	0.8 (1)
Sometimes	32.3 (43)
Rarely	7.5 (10)
Never	59.4 (79)
Total	**100.0 **(133)^*^

^*^Note: the total is not 146 as not all the clients of FSWs have regular partners.

**Table 2:** Clients using condoms consistently during sex with FSWs in the past 12 months in Tamil Nadu by selected background characteristics (*N* = 146).

Client characteristics (*P* value)	Number of clients using condoms consistently during sex (%)
Age (in years) (0.52)	
15–24	8 (53.3)
25–35	48 (51.1)
36–49	15 (40.5)
Years of education (0.13)	
0–5	6 (26.1)
6–10	39 (52.7)
11-12	15 (55.6)
>12	11 (50.0)
Occupation (0.18)	
Unemployed	9 (52.9)
Unskilled labor	18 (50.0)
Skilled labor	29 (59.2)
Service	9 (37.5)
Driving	6 (30.0)
Marital status (0.28)	
Unmarried	21 (58.3)
Married and living with spouse	49 (46.2)
Divorced/widowed/separated	1 (25.0)
Awareness of STIs (0.08)	
No	3 (23.1)
Yes	68 (51.1)
Knowledge of role of condom in preventing transmission of STIs (0.09)	
No	7 (31.8)
Yes	64 (51.6)
Use of condom during sex by peers (<0.001)	
No/do not know	14 (23.0)
Yes	57 (67.1)
Alcohol intake before going to FSW (0.04)	
Very often	43 (42.2)
Rarely	13 (50.0)
Never	15 (83.3)
Exposure to television (0.13)	
No	7 (33.3)
Yes	64 (51.2)
Total	**71 (48.6)**

Note: *P* values < 0.05 are significant.

**Table 3:** Logistic regression estimates of consistent condom usage with FSWs among clients of FSWs in the 12 months preceding the survey in Tamil Nadu (*N* = 146).

Explanatory variable	Unadjusted odds ratio (95% CI)	*P* value	Adjusted odds ratio (95% CI)	*P* value
Age group (in years)				
15–24^®^	1.00	—	1.00	—
25–35	0.91 (0.3–2.7)	0.87	0.49 (0.1–2.3)	0.37
36–49	0.60 (0.2–2.0)	0.40	0.61 (0.1–3.6)	0.58
Years of education				
0–5^®^	1.00	—	1.00	—
6–10	3.16 (1.1–8.9)	0.03∗	4.31 (0.9–19.5)	0.06
11-12	3.54 (1.1–11.8)	0.04∗	13.33 (2.1–85.8)	0.006∗∗
>12	2.83 (0.8–9.9)	0.10	10.82 (1.6–72.8)	0.01∗
Occupation				
Unemployed^®^	1.00	—	1.00	—
Unskilled labor	0.89 (0.3–2.8)	0.84	2.69 (0.5–16.1)	0.28
Skilled labor	1.29 (0.4–3.9)	0.65	4.42 (0.8–25.7)	0.10
Service	0.53 (0.2–1.9)	0.33	0.90 (0.1–5.8)	0.92
Driving	0.38 (0.1–1.5)	0.16	0.70 (0.1–4.8)	0.72
Marital status				
Unmarried^®^	1.00	—	1.00	—
Married and living with spouse	0.61 (0.3–1.3)	0.21	0.87 (0.3–3.1)	0.83
Divorced/widowed/separated	0.24 (0.0–2.5)	0.23	0.10 (0.00–4.7)	0.25
Awareness of STIs				
No^®^	1.00	—	1.00	—
Yes	3.49 (0.9–13.2)	0.07	1.90 (0.2–19.6)	0.59
Knowledge of role of condom in preventing transmission of STIs				
No^®^	1.00	—	1.00	—
Yes	2.29 (0.9–6.0)	0.09	0.79 (0.1–5.1)	0.81
Use of condom during sex by peers				
No/do not know^®^	1.00	—	1.00	—
Yes	6.83 (3.2–14.5)	<0.001∗∗∗	13.00 (4.9–34.3)	<0.001∗∗∗
Alcohol consumption when visiting FSWs				
Very often^®^	1.00	—	1.00	—
Rarely	1.37 (0.6–3.3)	0.47	1.34 (0.4–4.1)	0.62
Never	6.86 (1.9–25.2)	0.04∗	7.48 (1.3–41.8)	0.02∗
Exposure to television				
No^®^	1.00	—	1.00	—
Yes	2.10 (0.8–5.6)	0.14	3.71 (0.9–15.6)	0.07

Note: ^®^reference category.

^*^ *P* < 0.05, ^**^ *P* < 0.01, ^***^ *P* < 0.001.
